# STIM1 Regulates Platelet-Derived Growth Factor-Induced Migration and Ca^2+^ Influx in Human Airway Smooth Muscle Cells

**DOI:** 10.1371/journal.pone.0045056

**Published:** 2012-09-11

**Authors:** Nobukazu Suganuma, Satoru Ito, Hiromichi Aso, Masashi Kondo, Mitsuo Sato, Masahiro Sokabe, Yoshinori Hasegawa

**Affiliations:** 1 Department of Respiratory Medicine, Nagoya University Graduate School of Medicine, Nagoya, Japan; 2 Department of Physiology, Nagoya University Graduate School of Medicine, Nagoya, Japan; University of Debrecen, Hungary

## Abstract

It is suggested that migration of airway smooth muscle (ASM) cells plays an important role in the pathogenesis of airway remodeling in asthma. Increases in intracellular Ca^2+^ concentrations ([Ca^2+^]_i_) regulate most ASM cell functions related to asthma, such as contraction and proliferation. Recently, STIM1 was identified as a sarcoplasmic reticulum (SR) Ca^2+^ sensor that activates Orai1, the Ca^2+^ channel responsible for store-operated Ca^2+^ entry (SOCE). We investigated the role of STIM1 in [Ca^2+^]_i_ and cell migration induced by platelet-derived growth factor (PDGF)-BB in human ASM cells. Cell migration was assessed by a chemotaxis chamber assay. Human ASM cells express STIM1, STIM2, and Orai1 mRNAs. SOCE activated by thapsigargin, an inhibitor of SR Ca^2+^-ATPase, was significantly blocked by STIM1 siRNA and Orai1 siRNA but not by STIM2 siRNA. PDGF-BB induced a transient increase in [Ca^2+^]_i_ followed by sustained [Ca^2+^]_i_ elevation. Sustained increases in [Ca^2+^]_i_ due to PDGF-BB were significantly inhibited by a Ca^2+^ chelating agent EGTA or by siRNA for STIM1 or Orai1. The numbers of migrating cells were significantly increased by PDGF-BB treatment for 6 h. Knockdown of STIM1 and Orai1 by siRNA transfection inhibited PDGF-induced cell migration. Similarly, EGTA significantly inhibited PDGF-induced cell migration. In contrast, transfection with siRNA for STIM2 did not inhibit the sustained elevation of [Ca^2+^]_i_ or cell migration induced by PDGF-BB. These results demonstrate that STIM1 and Orai1 are essential for PDGF-induced cell migration and Ca^2+^ influx in human ASM cells. STIM1 could be an important molecule responsible for airway remodeling.

## Introduction

Airway remodeling due to repeated airway wall damage and repair plays an important role in the pathophysiology of severe asthma [1]. An increase of airway smooth muscle (ASM) mass due to proliferation and hypertrophy of ASM cells is one of the major pathological features of airway remodeling [1]. In addition, accumulating evidence suggests that ASM cell migration toward the airway epithelium in response to inflammatory mediators such as platelet-derived growth factor (PDGF) contributes to the airway remodeling [2]–[9]. As a result, the ASM layer in asthmatic patients is in close proximity to airway epithelial cells [6], [10], which may lead to increased airway hyperresponsiveness.

Intracellular free Ca^2+^ is a second messenger for ASM cell functions related to asthma, such as contraction, proliferation, and cytokine production [11]–[14]. Store-operated Ca^2+^ entry (SOCE), originally introduced as capacitative Ca^2+^ entry by Putney [15], is a ubiquitous Ca^2+^ influx pathway in various cell types including ASM cells [11], [16]–[18]. SOCE is activated by a fall in the Ca^2+^ concentration of the sarcoplasmic reticulum (SR) Ca^2+^ stores in muscle cells or endoplasmic reticulum (ER) in non-muscle cells through the binding of inositol–1,4,5-trisphosphate (IP_3_) to the IP_3_ receptor [18]. Importantly, SOCE closely links to the contraction and cell proliferation of ASM cells [11], [14], [19]–[21]. Stromal interaction molecule 1 (STIM1) was identified as a key molecule which senses Ca^2+^ concentrations within the SR and reports this information to Orai1, a Ca^2+^-permeable channel responsible for SOCE [22]–[26]. Peel et al. have demonstrated that SOCE is mediated by STIM1 and Orai1 in human ASM cells [27], [28]. However, whether STIM1 is involved in the mechanisms of ASM cell migration is still unknown.

This study was designed to investigate the role of STIM1 in the cell migration and the regulation of intracellular Ca^2+^ concentrations ([Ca^2+^]_i_) mediated by a strong chemoattractant, PDGF, in human ASM cells. We demonstrated that both STIM1 and Orai1 are essential for cell migration and elevation of [Ca^2+^]_i_ induced by PDGF in ASM cells.

## Materials and Methods

### Cell Culture

Primary cultures of normal human bronchial smooth muscle cells from multiple donors were obtained from Lonza (Walkersville, MD). The cells were maintained in culture medium containing 5% FBS, human recombinant epidermal growth factor (1 ng/ml), insulin (10 mg/ml), human recombinant fibroblast growth factor (2 ng/ml), gentamycin (50 mg/ml), and amphotericin B (0.05 mg/ml) (SmGM-2 BulletKit; Lonza) in an atmosphere of 5% CO_2_ and 95% air at 37°C [12], [29], [30].

### RT-PCR and Quantitative Real-Time PCR

Total cellular RNA was extracted using RNeasy Mini Kit (Qiagen, Hilden, Germany) [17]. RNA was reverse transcribed to cDNA using a Superscript III kit (Invitrogen, Carlsbad, CA). Polymerase chain reaction (PCR) amplification was performed with 35 cycles of denaturation at 94°C for 30 s, annealing at 60°C for 30 s, and extension at 72°C for 1 min. The sequences of the forward and reverse primers, respectively, were STIM1: 5′-CCAGAGCCTCAGCCATAGTC-3′ and 5′-CTTCAGCACAGTCCCTGTCA-3′, STIM2: 5′-GCTAAGGGAGGGAGCTGAAT-3′ and 5′-GCTAAGGGAGGGAGCTGAAT-3′, Orai1: 5′-TTCCTAGCTGAGGTGGTGCT-3′ and 5′-AATCCTCTTCCCTCCATGCT-3′ GAPDH: 5′-AACGGATTTGGTCGTATTGG-3′ and 5′-TGAGTCCTTCCACGATACCA-3′. Product sizes of the STIM1, STIM2, Orai1, and GAPDH were 481bp, 498bp, 483bp and 498bp, respectively.

Quantitative PCR was performed on a 7300 Real-Time PCR system (Applied Biosystems, Foster City, CA) using the 3-stage program parameters provided by the manufacturer: 2 min at 50°C, 10 min at 95°C, and then 40 cycles of 15 s at 95°C and 1 min at 60°C. Relative changes in each mRNA expression compared to an unstimulated control and normalized to GAPDH were quantified by the comparative Ct (2^−ddCt^) method using Microsoft Excel 2010 [31]. TaqMan Gene Expression Assays for STIM1 (cat# Hs00963373_m1), STIM2 (cat# Hs00956219_m1), Orai1 (cat# Hs00385627_m1), and GAPDH (Hs99999905_m1) genes (Applied Biosystems) in a reaction volume of 20 μL, including 50 ng cDNA were performed.

### Cell Transfections of siRNA

Targeting short interfering RNAs (siRNA) and the scrambled siRNA (negative control) were purchased from Invitrogen (Paisley, UK). Cells were transfected with 10 nM predesigned siRNA (Stealth Select RNAi) targeting STIM1, STIM2, and Orai1 or with 10 nM scrambled siRNA (negative control). Lipofectamine RNAiMAX (Invitrogen) was used as a transfection vector. To minimize the possibility of off target effects, three different siRNAs targeting either gene were used. Cells were used for PCR, Western blotting, Ca^2+^ measurement, and migration assays 48 h after siRNA transfection.

### Western Blotting

Protein concentrations of cellular lysates were measured by using a protein assay reagent kit (Bio-Rad, Hercules, CA). Equal amounts of lysates, adjusted for protein concentrations, were resolved by SDS-PAGE using a 5–20% linear gradient running gel (Wako, Osaka, Japan). Proteins were transferred to nitrocellulose membranes, and the membranes were blocked in 5% skim milk for 2 h at room temperature, followed by overnight incubation at 4°C with primary antibodies. The membranes were incubated for 1 h at room temperature with a sheep anti-mouse or donkey anti-rabbit secondary antibody. The primary antibodies used were a mouse anti-GOK/Stim1 antibody (BD Biosciences, CA), a rabbit anti-STIM2 antibody (Abcam, Tokyo, Japan), and a mouse anti-Orai1 antibody (Sigma-Aldrich, St. Louis, MO). A polyclonal anti-actin antibody (Sigma-Aldrich) was used for the loading control. Detection was performed with an Enhanced Chemiluminescence (ECL) kit (Amersham Biosciences, Piscataway, NJ) [12], [31].

### Measurement of Intracellular Ca^2+^ Concentrations

Cells (approximately 50% confluence) grown on glass coverslips (Lab-Tek; Nunc, Rochester, NY) were treated with 3 µM fura-2/AM (Dojin, Kumamoto, Japan) for 25 min at 37°C in normal physiological solution containing (in mM): NaCl 145, KCl 5, CaCl_2_ 2, MgCl_2_ 1, glucose 10, and HEPES 10 (pH 7.40). After the cells were washed with normal physiological solution, the [Ca^2+^]_i_ was assessed by the fluorescence of fura-2 using a fluorescence microscope (Fluor20; Nikon, Tokyo, Japan) at room temperature. Data were analyzed using a digital fluorescence imaging system (Aquacosmos; Hamamatsu Photonics, Hamamatsu, Japan). The excitation wavelengths were set at 340 and 380 nm, and the emission was collected at 510 nm by a photomultiplier. The intensity of the fura-2 fluorescence due to excitation at 340 nm (F_340_) and 380 nm (F_380_) was measured after subtraction of the background fluorescence, and the ratio of F_340_ to F_380_ (F_340_/F_380_ ratio) was used as an indicator of the relative level of [Ca^2+^]_i_ [17], [29]. The fura-2 fluorescence of 7–10 cells per field was analyzed using individual regions of interest for each experiment and “n” refers to numbers of experiments tested.

### Cell Migration Assay

Cell migration was measured using a modified Boyden chamber (Chemotaxicell; Kurabo, Osaka, Japan). Chambers with 8-μm pores were coated with type-1 collagen (Nitta Gelatin Inc., Osaka, Japan). Confluent ASM cells were brought to a quiescent state overnight by incubation in DMEM/F-12 cell culture medium (Invitrogen) containing 0.1% FBS before being used in a migration assay. Cells (2×10^4^) suspended in 400 μL of DMEM/F-12 containing 0.1% FBS were placed in the upper chamber. PDGF-BB (Sigma-Aldrich) dissolved in DMEM/F-12 containing 0.1% FBS was inserted in the wells of the lower chamber. The cells were transferred to the upper wells, and after incubation for 6 h at 37°C in a 5% CO_2_ incubator, the non-migrated cells on the upper surface of the filter were scraped off with a cotton-tipped applicator. The migrated cells were fixed and stained with Diff-Quik (Sysmex, Kobe, Japan) and mounted onto glass slides. Cells in five fields per chamber were counted under a light microscope (x200). In the Results section, “n” refers to numbers of experiments tested. Each experimental condition was tested in duplicate. Solvents did not affect cell migration at the concentrations used (≤0.1%/vol.).

### Statistical Analysis

All data are expressed as means ± SD. Analysis of variance followed by the Bonferroni test for post hoc analysis or *t*-test was used to evaluate the statistical significance (SigmaPlot11.0; Systat Software Inc., San Jose, CA). P<0.05 was considered statistically significant.

## Results

### Expression of STIM1, STIM2, and Orai1 in Human Airway Smooth Muscle Cells

**Figure 1:**
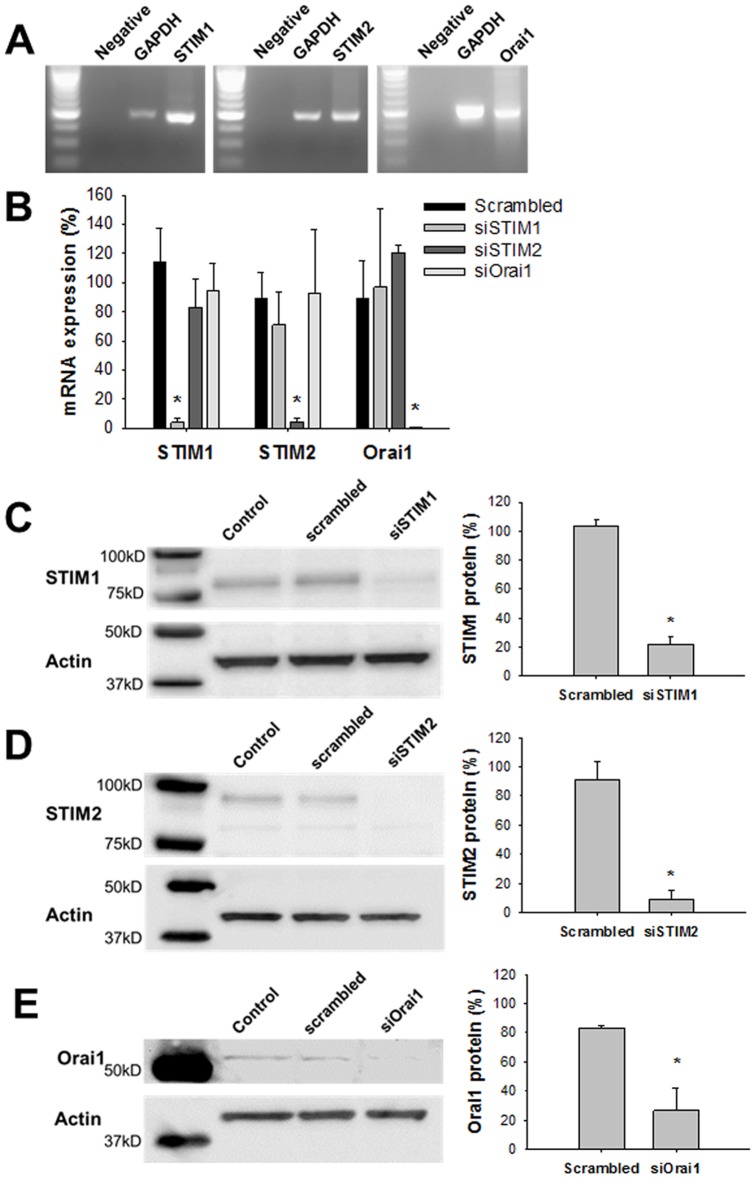
Expression of STIM1, STIM2, and Orai1. **A**: Expression of STIM1, STIM2, Orai1, and GAPDH mRNAs detected by RT-PCR in human airway smooth muscle (ASM) cells is shown. Negative indicates a negative control. The product sizes for STIM1, STIM2, Orai1, and GAPDH were 481bp, 498bp, 483bp, and 498bp, respectively. **B**: Effects of siRNA-targeted knockdown of STIM1, STIM2, and Orai1 mRNAs on the change in mRNA expression over control normalized to the reference gene GAPDH are shown (n = 4). Changes in mRNA expression were assessed by quantitative real-time PCR. Effects of siRNA transfection targeting STIM1 (siSTIM1) (**C**), STIM2 (siSTIM2) (**D**), and Orai1 (siOrai1) (**E**) on changes in protein levels were assessed by Western blot. STIM1, STIM2, and Orai1 protein levels expressed as the target protein/actin ratio in the cells transfected with siSTIM1 (**C**), siSTIM2 (**D**), or siOrai1 (**E**) and scrambled siRNA (negative control) are compared (n = 3). The control value without siRNA transfection is defined as 100%. *Significantly different from the values of the scrambled siRNA condition (P<0.05). Bars represent means ± SD.

We initially measured the expression of STIM1, STIM2, another STIM protein similar in structure to STIM1 [32], and Orai1 in human ASM cells. Expression of STIM1, STIM2, and Orai1 mRNAs assessed by RT-PCR is shown in Figure 1A. Next, the cells were transfected with siRNA sequences for STIM1 (siSTIM1), STIM2 (siSTIM2), or Orai1 (siOrai1). Real-time quantitative PCR data showed that transfection of siSTIM1, siSTIM2, and siOrai1 induced a large decrease in mRNA levels of target genes without altering mRNA levels of non-target genes (Figure 1B). Average mRNA levels for STIM1, STIM2, and Orai1 normalized to GAPDH in the cells transfected with siRNAs were 3.9%, 4.2%, and 0.6%, respectively (n = 4, P<0.001 vs. scrambled siRNA) (Figure 1B). Three different siRNAs targeting the same gene were tested and gave similar mRNA expression results (data not shown). These findings demonstrate that the siRNAs used in the present study had no off-target effects. The effects of siRNA transfection on protein levels of STIM1, STIM2, and Orai1 were assessed by Western blotting. Transfection with siRNAs for STIM1, STIM2, and Orai1 inhibited protein expression of STIM1, STIM2, and Orai1, respectively (Figure 1C, 1D, 1E). STIM1 protein expression as assessed by the STIM1/actin ratio was significantly lower in the cells transfected with siSTIM1 than the control cells transfected with scrambled siRNA (n = 3, P<0.001) (Figure 1C). Similarly, the STIM2/actin ratio and Orai1/actin ratio were significantly lower in the cells transfected with siSTIM2 (n = 3, P<0.001) (Figure 1D) and siOrai1 (n = 3, P<0.001) (Figure 1E) than the control cells transfected with scrambled siRNA.

### Store-Operated Ca^2+^ Entry Induced by Thapsigargin

**Figure 2:**
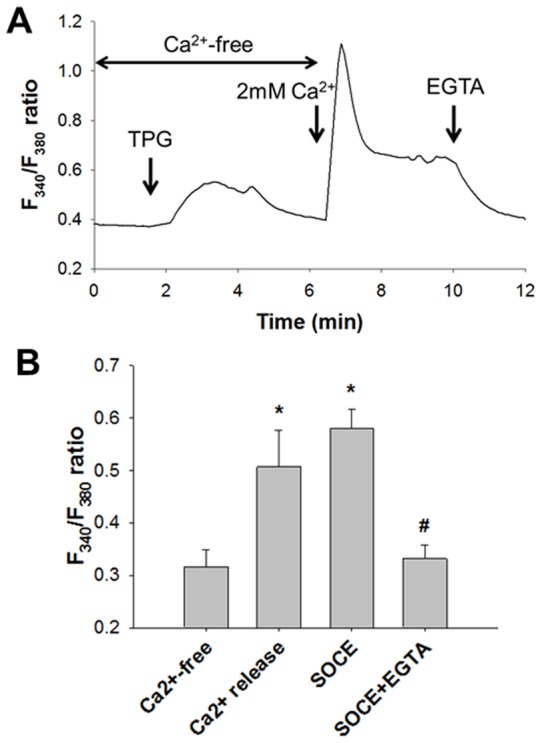
Store-Operated Ca^2+^ Entry Induced by Thapsigargin. Store-operated Ca^2+^ entry (SOCE) activated by thapsigargin. **A**: Representative traces of the F_340_/F_380_ ratio, a measure of intracellular Ca^2+^ concentrations ([Ca^2+^]_i_), by 5 μM thapsigargin (TPG). After the cells were treated with 5 μM thapsigargin in the nominally Ca^2+^-free solution, 2 mM Ca^2+^ was added to the solution. At the end, 2 mM EGTA was added. **B**: The F_340_/F_380_ ratios in nominally Ca^2+^-free solution (Ca^2+^-free), in response to 5 μM thapsigargin in the Ca^2+^-free solution due to Ca^2+^ release, in the normal solution containing 2 mM Ca^2+^ with thapsigargin due to SOCE (the plateau phase), and in the normal solution with thapsigargin and 2 mM EGTA (SOCE+EGTA). Bars represent the means ± SD (n = 6). Significantly different from values in the Ca^2+^-free solution (*) and of SOCE (#) (P<0.05).

It is established that thapsigargin, an inhibitor of SR Ca^2+^-ATPase (SERCA), induces SOCE by depleting SR Ca^2+^ stores in ASM cells [11]. When 5 μM thapsigargin (Calbiochem, La Jolla, CA) was applied to the cells in the nominally Ca^2+^-free solution, a transient increase in the F_340_/F_380_ ratio, a measure of [Ca^2+^]_i_, due to Ca^2+^ release from the SR Ca^2+^ stores was observed (Figure 2A and 2B). When 2 mM Ca^2+^ was added to the extracellular solution, the F_340_/F_380_ ratio was quickly increased to approximately 1.0 and then dropped to 0.6–0.7 and sustained (Figure 2A). This sustained increase (plateau phases) in the F_340_/F_380_ ratio was abolished by replacing the exracellular solution with Ca^2+^-free solution or application of 2 mM EGTA (n = 6, P<0.001) (Figure 2A and 2B), demonstrating that the second phase was due to sustained activation of SOCE.

### Role of STIM1 and Orai1 in Store-Operated Ca^2+^ Entry

**Figure 3:**
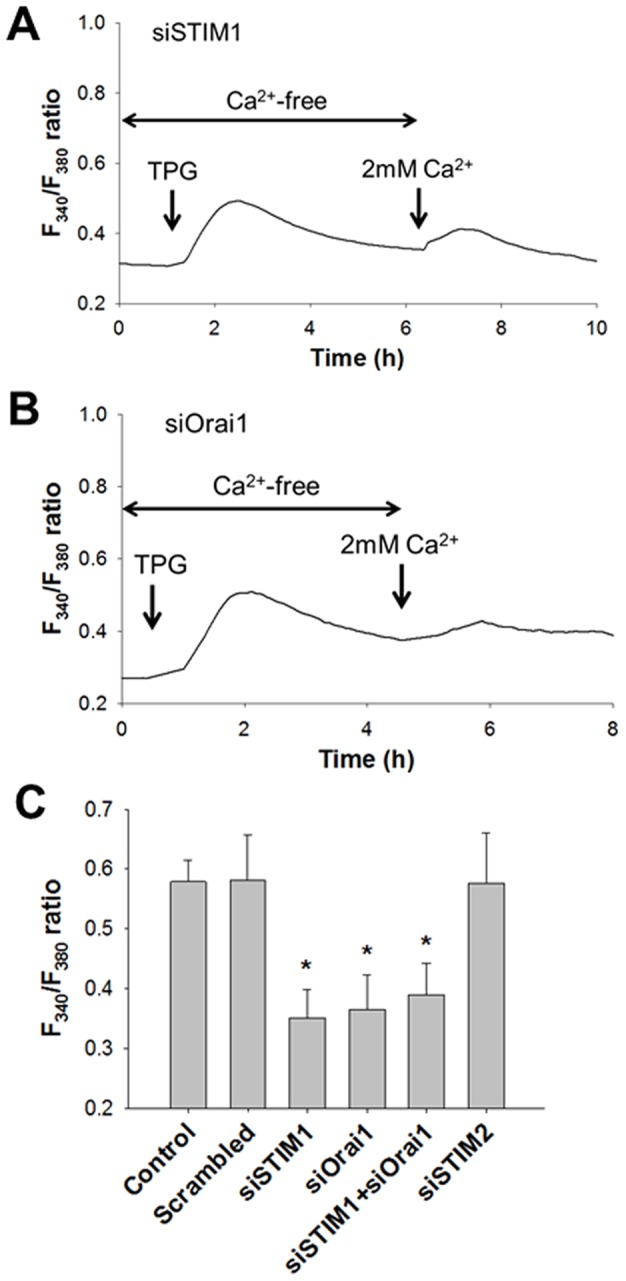
Role of STIM1 and Orai1 in Store-Operated Ca^2+^ Entry. Roles of STIM1 and Orai1 in SOCE. Representative changes in the F_340_/F_380_ ratio due to 5 μM thapsigargin (TPG) in cells transfected with siRNA targeting STIM1 (**A**) and Orai1 (**B**). After the cells were treated with thapsigargin in the nominally Ca^2+^-free solution, 2 mM Ca^2+^ was added to the solution. **C**: The F_340_/F_380_ ratios in response to 5 μM thapsigargin in the normal solution due to SOCE with or without (control) siRNA treatment. The cells transfected with scrambled siRNA, siSTIM1, siOrail, both siSTIM1 and Orai1, or siSTIM1. Bars represent the means ± SD (n = 6). *Significantly different from the values of the cells transfected with scrambled siRNA (P<0.05).

Next, we assessed the role of STIM1, STIM2, and Orai1 in SOCE in human ASM cells. The effects of a knockdown of STIM1, STIM2, and Orai1 genes with siRNA on SOCE were examined. Representative records of the effects of 5 μM thapsigargin on the F_340_/F_380_ ratio in cells transfected with siSTIM1 and siOrai1 are shown in Figure 3A and 3B, respectively. The transient increase in F_340_/F_380_ ratio in the nominally Ca^2+^-free solution due to Ca^2+^ release was still observed in the cells transfected with siSTIM1 (Figure 3A) or siOrai1 (Figure 3B). In contrast, the second increase in F_340_/F_380_ ratio due to SOCE was strongly inhibited by siSTIM1 transfection (Figure 3A). The rapid increase in the F_340_/F_380_ ratio was not observed (Figure 3A) and the F_340_/F_380_ ratio of the plateau phase was significantly lower in siSTIM1-transfected cells (n = 6, P<0.001) (Figure 3C). Similarly, SOCE activated by 5 μM thapsigargin was significantly inhibited in the cells transfected with siOrai1 (n = 6, P<0.001) (Figure 3B and 3C). Moreover, simultaneous transfection with siSTIM1 and siOrai1 also significantly inhibited SOCE by 5 μM thapsigargin (n = 6, P<0.001) (Figure 3C). The increase in the F_340_/F_380_ due to SOCE (plateau phase) was not affected by siSTIM2 or scrambled siRNA (n = 6) (Figure 3C).

### Effects of PDGF on Intracellular Ca^2+^ Concentrations

**Figure 4:**
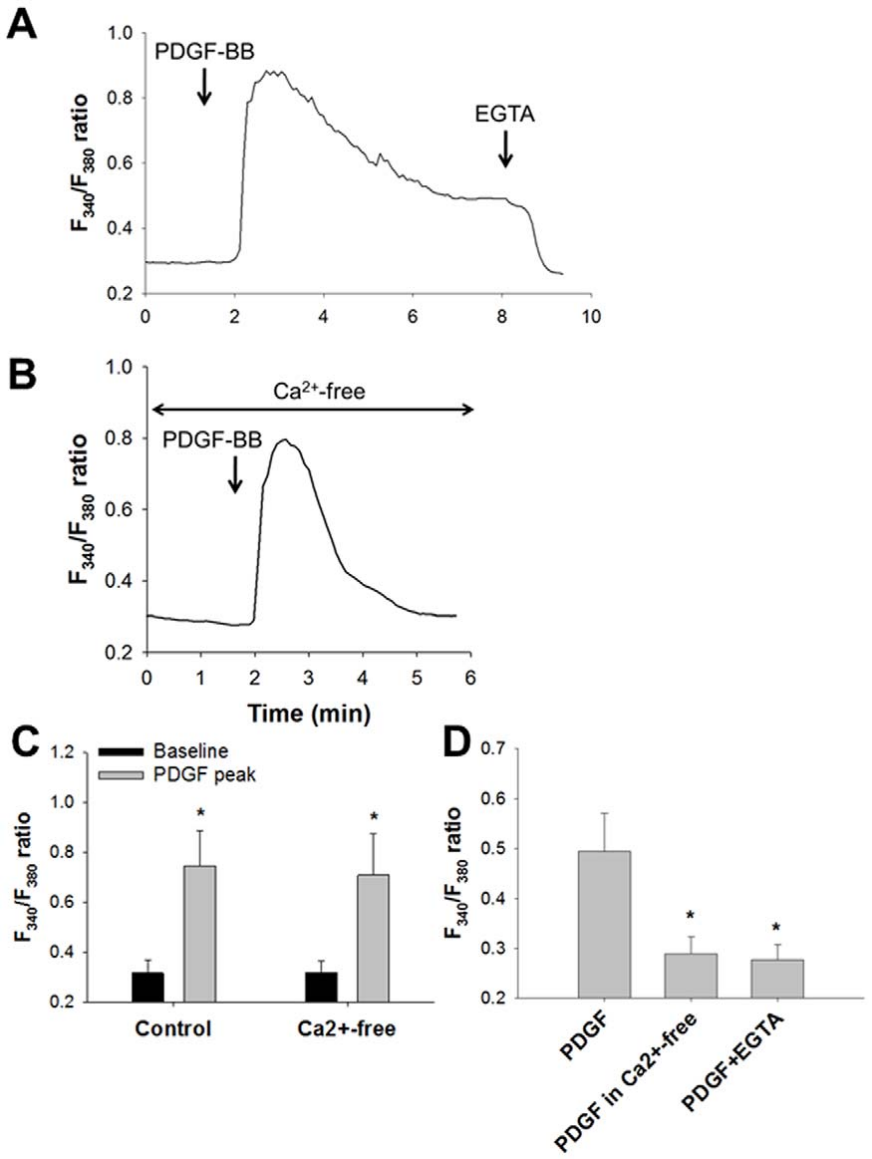
Effects of PDGF on Intracellular Ca^2+^ Concentrations. Effects of PDGF-BB on [Ca^2+^]_i_. Representative changes in the F_340_/F_380_ ratio with 10 ng/mL PDGF-BB in the normal solution (**A**) or in the nominally Ca^2+^-free solution (**B**). **C**: The peak F_340_/F_380_ ratios in response to PDGF-BB in the normal solution (control) or the nominally Ca^2+^-free solution. Bars represent the means ± SD (n = 6). *Significantly different from the baseline values without PDGF-BB treatment (P<0.05). **D**: The sustained F_340_/F_380_ ratios (plateau phases) in response to PDGF-BB in the normal solution (control) or the nominally Ca^2+^-free solution. Effects of EGTA (2 mM) on sustained increase in the F_340_/F_380_ ratio with 10 ng/mL PDGF-BB are also shown. Bars represent the means ± SD (n = 6). *Significantly different from the values with PDGF-BB alone (P<0.05).

Effects of PDGF-BB on [Ca^2+^]_i_ were investigated. Application of PDGF-BB (10 ng/mL) to the normal physiological solution containing 2 mM Ca^2+^ induced a transient increase in the F_340_/F_380_ ratio, followed by a sustained increase in the F_340_/F_380_ ratio (Figure 4A). Application of EGTA (2 mM) abolished the sustained increase in the F_340_/F_380_ ratio by PDGF-BB (Figure 4A). PDGF-BB transiently increased the F_340_/F_380_ ratio in nominally Ca^2+^-free solution (Figure 4B). However, the increase in the F_340_/F_380_ ratio by application of PDGF-BB returned to the baseline level in Ca^2+^-free solution (Figure 4B). There was no significant difference between peak F_340_/F_380_ ratios elicited by PDGF-BB in the cells in the normal (control) and nominally Ca^2+^-free solutions (n = 6) (Figure 4C). The sustained increases in the F_340_/F_380_ ratio by application of PDGF-BB were significantly lower in the nominally Ca^2+^-free solution or the normal solution with 2 mM EGTA than those in the normal solution (control) (n = 6, P<0.001) (Figure 4D). These results indicate that the initial transient increase and the subsequent sustained phase (plateau phase) are due to Ca^2+^ release from the SR and Ca^2+^ influx from the extracellular side, respectively.

### Role of STIM1 in PDGF-Induced Intracellular Ca^2+^ Elevation

**Figure 5:**
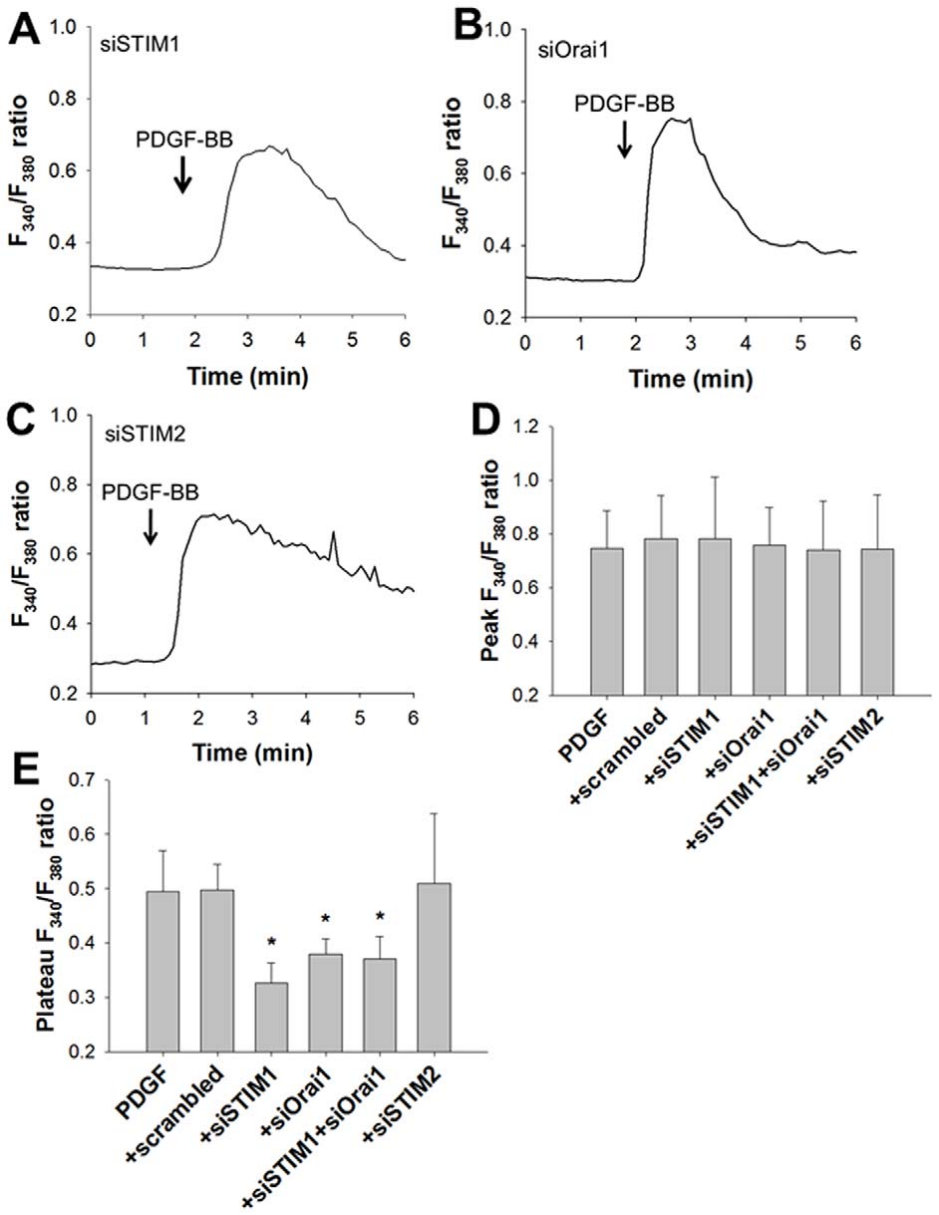
Role of STIM1 in PDGF-Induced Intracellular Ca^2+^ Elevation. Roles of STIM1 and Orai1 in [Ca^2+^]_i_ elevation induced by PDGF-BB. Representative changes in the F_340_/F_380_ ratios with 10 ng/mL PDGF-BB in the cells transfected with siSTIM1 (**A**) and siOrai1 (**B**), and siSTIM2 (**C**) are shown. Transient (peak) (**D**) and sustained increases (plateau phase) (**E**) in the F_340_/F_380_ ratio in response to PDGF-BB with or without (control) siRNA treatment are compared. Bars represent the means ± SD (n = 6). *Significantly different from the values of the control cells treated with 10 ng/mL PDGF-BB plus scrambled siRNA (P<0.05).

We next evaluated whether STIM1 and Orai1 mediate increases in [Ca^2+^]_i_ by PDGF-BB. The transient increase in the F_340_/F_380_ ratio induced by PDGF-BB was still observed (Figure 5A) but the F_340_/F_380_ ratio gradually decreased close to the baseline level within 5 min after PDGF-BB application in the cells transfected with siSTIM1 (Figure 5A). Similarly, PDGF-BB induced the transient increase in the F_340_/F_380_ ratio in the cells transfected with siOrai1 (Figure 5B). In contrast, the PDGF-BB-induced elevation of the F_340_/F_380_ ratio was not reduced by siSTIM2 transfection (Figure 5C). The peak F_340_/F_380_ ratio was not affected by transfection with siSTIM1, siOrai1, or siSTIM2 (n = 6) (Figure 5D). The F_340_/F_380_ ratio in the plateau phase was significantly lower in the siSTIM1- or siOrai1-transfected cells than in the cells transfected with scrambled siRNA (negative control) (n = 6, P<0.001) (Figure 5E). Simultaneous transfection with siSTIM1 and siOrai1 also significantly inhibited the PDGF-BB-induced increase in the F_340_/F_380_ ratio (n = 6, P<0.05) (Figure 5E) without affecting the peak F_340_/F_380_ ratio (Figure 5D), similar to transfection with siSTIM1 or siOrai1 alone. In contrast, the PDGF-BB-induced elevation of the F_340_/F_380_ ratio was not inhibited by siSTIM2 (n = 6) (Figure 5E).

### Roles of STIM1 and Orai1 in Cell Migration Induced by PDGF

**Figure 6:**
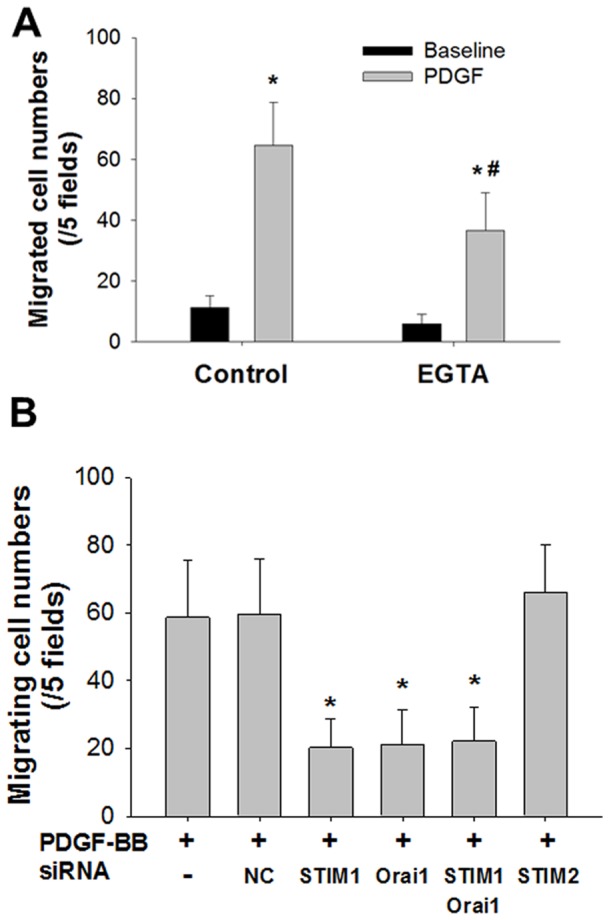
Roles of STIM1 and Orai1 in Cell Migration Induced by PDGF. Roles of STIM1 and Orai1 in cell migration induced by PDGF-BB (10 ng/mL, 6 h) are shown. Cell migration was assessed by a chemotaxis assay. **A**: Effects of PDGF-BB on migrated cell numbers with or without (control) 1 mM EGTA treatment (n = 6). Baseline (black column) denotes the time-matched number of cells that migrated without PDGF-BB treatment. Significantly different from the values of the baseline (*) and by PDGF-BB alone (#) (P<0.05). **B**: Effects of siRNA treatment targeting STIM1, STIM2, Orai1, and both STIM1 and Orai1 on migrating cell numbers induced by PDGF-BB (n = 6). *Significantly different from the values of the time-matched control cells treated with PDGF-BB plus scrambled siRNA (negative control, NC) (P<0.05). Bars represent means ± SD.

The roles of STIM1 and Orai1 in PDGF-induced cell migration were investigated using a chemotaxis assay. Migrating cell numbers were significantly increased by treatment with PDGF-BB (10 ng/mL, 6 h) compared with time-matched control cell cultures (n = 6, P<0.001) (Figure 6A). Moreover, cell migration induced by PDGF-BB (10 ng/mL, 6 h) was also significantly inhibited by 1 mM EGTA (n = 6, P<0.001) (Figure 6A). There was no significant difference in baseline cell migration (in cell culture media with 0.1% FBS, 6 h) between the control and EGTA-treated cells (Figure 6A).

Next, cells were transfected with siRNAs targeting STIM1, STIM2, Orai1, or the negative control (scrambled siRNA). Transfection with siSTIM1, siOrai1, or both siSTIM1 and siOrai1 significantly inhibited PDGF-BB-induced cell migration (n = 6, P<0.001 vs. scrambled siRNA) (Figure 6B). In contrast, siSTIM2 or scrambled siRNA did not affect the PDGF-induced cell migration (n = 6) (Figure 6B).

## Discussion

This study highlights the novel role of STIM1 in migration of ASM cells. The main findings are that (1) SOCE activation by thapsigargin was inhibited by siRNAs targeting STIM1 and Orai1, (2) a sustained increase in [Ca^2+^]_i_ induced by PDGF-BB was also inhibited by siSTIM1 and siOrai1, (3) STIM1 and Orai1 were essential for PDGF-induced ASM cell migration, and (4) STIM2 was not involved in these mechanisms. To our knowledge, this is the first report which demonstrates an essential role of STIM1 and Orai1 in migration and increases in [Ca^2+^]_i_ induced by PDGF in human ASM cells.

STIM1 was identified as the key molecule for SOCE [23], [25]. STIM1 predominantly exists in the SR or ER and has its N-terminal sensing Ca^2+^ domain in the SR/ER lumen and its C-terminal Orai1 coupling site in the cytosol [32]. When the amount of Ca^2+^ contents within the SR or ER is decreased, STIM1 rapidly forms oligomers and activates Orai1, a highly Ca^2+^-selective plasma-membrane cation channel [24], [26]. It has been reported that STIM1 and Orai1 regulate SOCE in human and rat ASM cells [21], [27], [28]. In our results, SOCE activated by thapsigargin was inhibited by siSTIM1 and siOrai1. In contrast, STIM2 is not involved in the regulation of SOCE in human ASM cells despite its expression (Figures 1 and 3). These are consistent with the findings reported by Peel et al. [27]. In human myoblasts, STIM2 regulates SOCE similarly to STIM1 [33]. Thus, the discrepancy in the role of STIM2 in the regulation of SOCE arises from the difference in cell types.

In the present study, increases in [Ca^2+^]_i_ due to PDGF-BB were significantly inhibited by knockdown of STIM1 and Orai1 with siRNA in human ASM cells (Figure 5). PDGF binds to PDGF receptors, members of receptor tyrosine kinases, which involve α and β subtypes [34]. PDGF-BB activates α and β receptors both of which are expressed in human ASM cells [34], [35]. It is known that PDGF receptors cause phospholipase Cγ activation and intracellular Ca^2+^ mobilization [34], [36]. Indeed, stimulation of human ASM cells by PDGF-BB transiently elicited elevation of [Ca^2+^]_i_ even in the Ca^2+^-free solution (Figure 4), demonstrating that PDGF-BB induces Ca^2+^ release from the SR. This transient increase of [Ca^2+^]_i_ was still observed in the cells transfected with siSTIM1 or siOrai1 (Figure 5). Therefore, STIM1 and Orai1 are not involved in the mechanisms of Ca^2+^ release from the SR via IP_3_ receptors. In contrast, the sustained increase of [Ca^2+^]_i_ due to PDGF-BB was abolished in the Ca^2+^-free solution and largely inhibited by siSTIM1 and siOrai1 (Figures 4 and 5). These findings demonstrate that STIM1 and Orai1 regulate the Ca^2+^ influx pathway for the sustained [Ca^2+^]_i_ elevation by PDGF-BB in human ASM cells. It has been reported that SOCE mediated by STIM1/Orai1 is crucial in the PDGF-induced increase of [Ca^2+^]_i_ in vascular smooth muscle cells [37]–[39], consistent with our findings in ASM cells.

Recently, several reports have shown that STIM1 and Orai1 are also involved in mechanisms of Ca^2+^ influx independent of Ca^2+^ stores. Xiao et al. demonstrated that heating to above 40°C induces clustering of STIM1 without depleting Ca^2+^ stores in Jurkat T cells [40]. Following cooling the cell off to 25°C, Orai1 was activated [40]. However, in our experimental conditions, it is unlikely that such temperature change-dependent activation of STIM1 and Orai1 is involved in the PDGF-induced Ca^2+^ influx. In a study by Liu et al. [41], Ca^2+^ influx via reverse mode Na^+^/Ca^2+^ exchange (NCX) was regulated by SR Ca^2+^ store depletion and STIM1 in human ASM cells. In their report, the histamine-induced increase of [Ca^2+^]_i_ was partially inhibited by the reverse mode NCX inhibitor KB-R7943 [41]. In our preliminary results, PDGF-induced migration of human ASM cells was not significantly inhibited by KB-R7943 (data not shown). These observations suggest that contribution of the reverse mode NCX to PDGF-induced Ca^2+^ influx is much less than that of Orai1 in ASM cells. In another report by Feng et al. [42], Orai1 was activated by Secretory Pathway Ca^2+^-ATPase 2 (SPCA2) independently of ER Ca^2+^ stores or STIM1 in breast cancer cells. By contrast to their results, we demonstrated that siOrai1 blocked the thapsigargin-induced SOCE and PDGF-induced [Ca^2+^]_i_ elevation (Figures 3 and 5). Moreover, simultaneous transfection with siSTIM1 and siOrai1 did not have additive effects on the PDGF-induced [Ca^2+^]_i_ elevation (Figure 5). Therefore, SPCA2-dependent, STIM1-independent Orai1 activation is not likely involved in the Ca^2+^ influx by PDGF-BB. Taken together, the PDGF-induced increase of [Ca^2+^]_i_ is mostly via SOCE in human ASM cells. Nevertheless, possible involvement of store-independent mechanisms in PDGF-induced [Ca^2+^]_i_ elevation cannot be ruled out.

Activation of PDGF receptors strongly promotes migration of ASM cells [2], [7], [34]. We found that transfection with siSTIM1, siOrai1, or both reduced the PDGF-induced migration of human ASM cells as assessed by the chemotaxis assay (Figure 6). It was determined that STIM1 and Orai1 regulate PDGF-evoked migration in a wound-healing assay using vascular smooth muscle cells [37], [39]. Furthermore, both STIM1 and Orai1 were implicated in cell migration in breast cancer cells and cervical cancer cells [43], [44]. Zou et al. demonstrated the involvement of STIM1 and Orai1 in the cell proliferation evoked by PDGF-BB in rat ASM cells [21]. These previous findings using different cell types and species also support our results. Taken together, STIM1 and Orai1 regulate PDGF-induced migration of human ASM cells. In contrast, STIM2 does not contribute to the migration or [Ca^2+^]_i_ elevation evoked by PDGF-BB in human ASM cells.

Migration of ASM cells is not only essential for development of hollow airways and the respiratory system but also important for airway remodeling in asthma [3], [4]. Nevertheless, the mechanisms of ASM cell migration are not fully elucidated yet. It has been proposed that the dynamics of the cytoskeleton and multiple signal transduction pathways, including intracellular Ca^2+^ signaling, are involved in processes of cell motility and migration [3], [36], [45], [46]. It is well established that activation of myosin light-chain kinase (MLCK) and subsequent myosin light chain phosphorylation is the main downstream pathway of [Ca^2+^]_i_ elevation in smooth muscle contraction [47]. In contrast, Carlin et al. demonstrated that PDGF-induced cell migration was not inhibited by MLCK inhibitors in human ASM cells [3]. Essentially the same results were observed in our preliminary experiments (data not shown). Thus, it is unlikely that MLCK is involved in the mechanism of ASM cell migration enhanced by PDGF. Further studies are necessary to identify the downstream pathways of SOCE in PDGF-induced ASM cell migration.

Because SOCE is a major influx pathway both in muscle and non-muscle cells, STIM1 and Orai1 proteins play critical roles in homeostasis of the immune system and normal development. Mutations in STIM1 and Orai1 genes are clinically characterized by severe immunodeficiency and congenital myopathy in human patients [48]. Mice lacking STIM1 or Orai1 gene die perinatally of unknown causes [48]. On the other hand, involvement of STIM1 and Orai1 in the pathogenesis of several diseases has also been reported [48]. Baba et al. reported that STIM1 is essential for mast cell activation and immunoglobulin E-mediated anaphylactic responses in mice [49]. Another possibility is that SOCE mediated by STIM1 and Orai1 is involved in the pathophysiology of cardiovascular diseases. In rat carotid artery, vascular smooth muscle cell proliferation in vitro and neointima formation after balloon injury in vivo were significantly inhibited by a knockdown of the STIM1 gene [50]. Bisaillon et al. demonstrated that mRNA levels of STIM1 and Orai1 were upregulated in a balloon-injured carotid artery compared with the control in rat models [37]. Therefore, SOCE is likely to contribute to vascular remodeling. ASM cells are the main effector cells of airway narrowing in asthma [51]. Recently, Sathish et al. found that both STIM1 and Orai1 are upregulated by TNF-α in human ASM cells [52]. Because the present and previous results have demonstrated that SOCE tightly regulates the contraction, proliferation, and migration of ASM cells [11], [14], [19], [21], STIM1 and Orai1 may be involved in mechanisms of the pathophysiology of airway diseases such as asthma and COPD.

In summary, STIM1 and Orai1, key molecules for SOCE, regulate PDGF-induced migration and Ca^2+^ influx of human ASM cells. Our findings suggest that STIM1 and Orai1 may be important molecules responsible for airway remodeling in asthma.
